# Evaluating the use of vision-face-arm-speech-time in stroke admissions: A comparative study with national institutes of health stroke scale and orthoptist assessment in an emergency department

**DOI:** 10.1177/02692155261450137

**Published:** 2026-05-14

**Authors:** Muath Alobaida, Deirdre A. Lane, Stephanie L. Harrison, Gregory Y. H. Lip, Fiona J. Rowe

**Affiliations:** 1 Liverpool Centre for Cardiovascular Science, 223914 University of Liverpool, Liverpool John Moores University and Liverpool Heart & Chest Hospital, Liverpool, UK; 2 Department of Cardiovascular and Metabolic Medicine, Institute of Life Course and Medical Sciences, University of Liverpool, Liverpool, UK; 3 Department of Basic Science, Prince Sultan Bin Abdulaziz College for Emergency Medical Services, King Saud University, Riyadh, Saudi Arabia; 4 Danish Center for Health Services, Department of Clinical Medicine, Aalborg University, Aalborg, Denmark; 5 Registry of Senior Australians, South Australian Health and Medical Research Institute, Adelaide, Australia; 6 223914 Institute of Population Health, University of Liverpool, Liverpool, UK

**Keywords:** Stroke, vision, visual impairment, V-FAST, National Institutes of Health Stroke Scale, emergency department, eye movements, visual fields, visual neglect, screening

## Abstract

**Objectives:**

Visual problems are common in acute stroke, however no standardised vision screening tool is routinely used in pre-hospital or emergency department settings. This study evaluated the V-FAST (vision-face-arm-speech-time) checklist for detecting visual impairment in patients admitted with suspected stroke.

**Design:**

Prospective, service evaluation study.

**Setting:**

One emergency department.

**Participants:**

About 282 consecutive patients with suspected stroke.

**Main measures:**

All patients were assessed with the National Institutes of Health Stroke Scale, V-FAST checklist, and reference standard orthoptic assessment. Main outcomes included visual impairment detection rates and diagnostic performance (sensitivity, specificity, predictive values, and agreement).

**Results:**

Of 282 screened patients (mean (SD) age 73 (11) years; 51.5% females), 158 (56.2%) had stroke confirmed. Among these, visual impairment was detected in 64.0% by orthoptists, 34.0% by the National Institutes of Health Stroke Scale (κ = 0.22), and 47.0% by V-FAST (κ=0.34). In FAST-positive strokes, National Institutes of Health Stroke Scale and V-FAST detected visual impairment in 83.3% and 76.0%, respectively; in FAST-negative strokes, detection was 16.7% and 24.0%, respectively, compared to orthoptic assessments. V-FAST showed better diagnostic performance than National Institutes of Health Stroke Scale, with sensitivity, specificity, positive/negative predictive values and kappa of 78.7%, 97.2%, 83.6%, 90.0%, and 0.79, respectively.

**Conclusion:**

The V-FAST checklist demonstrates fair agreement with orthoptist assessment and complements the National Institutes of Health Stroke Scale for detecting visual impairment in stroke admissions in the emergency department, with particular benefit in FAST-negative strokes. These findings support integrating a standardised screening checklist for visual impairment detection into emergency department stroke pathways.

## Introduction

Visual impairment is a common post-stroke complication, affecting up to 73% of survivors and occurring in 60% of acute cases,^1^ which can significantly impact recovery and quality of life.^2,3^ Traditional stroke assessments, such as face, arm, speech, time (FAST), often fail to detect visual impairments, particularly in patients with posterior circulation strokes where visual symptoms may predominate. Although the BE-FAST tool adds “balance” and “eyes” to expand symptom detection,^4^ BE-FAST lacks comprehensive evaluation for visual inattention or eye movements, limiting its utility for identifying various types of visual impairment and FAST-negative strokes in hyperacute settings.

The Vision-FAST (V-FAST) tool was developed as an adjunct to FAST to enhance early detection of stroke-related visual impairments by ambulance services. Unlike BE-FAST,^4^ V-FAST incorporates rapid screening of reading, visual fields, eye movements, extinction and a visual symptom checklist.^5^ A pilot study of 43 patients with suspected stroke in pre-hospital setting demonstrated V-FAST may improve detection of visual symptoms in both FAST-positive and FAST-negative presentations.^5^ However, while tools like the Visual Impairment Screening Assessment exist for stroke unit use,^6^ no standardised tool is routinely used for visual screening in hyperacute settings such as emergency department.^5^

This study aimed to validate V-FAST in an emergency department setting by comparing its performance against the National Institutes for Health Stroke Scale, a widely used stroke severity scale, and orthoptist evaluations (reference standard assessment) in detecting visual impairment in patients with suspected stroke.

## Methods

This study was conducted as a service evaluation and received regulatory approval from the local NHS Trust (Ref: SE 234 22 23). In line with UK Health Research Authority guidance, separate research ethics committee approval or individual patient consent was not required.

### Study design and assessments

This prospective service evaluation included all consecutive adults (≥18 years) with suspected stroke admitted to a single emergency department in North-West England from 3 April 2023 to 31 December 2023. To ensure full case capture, no exclusion criteria were applied (Supplemental Figure 1). V-FAST and National Institutes of Health Stroke Scale assessments were performed prospectively as part of standard clinical care. Baseline clinical data were collected anonymously from electronic health records for analysis. V-FAST was developed by the VISION research team at the University of Liverpool for use in pre-hospital settings.^5^ Diagnostic accuracy outcomes were selected and reported in line with the Standards for Reporting Diagnostic Accuracy Studies 2015 guidelines to ensure methodological rigour and transparency.

In the recruiting hospital, National Institutes of Health Stroke Scale screening is routinely performed by stroke nurses, who act as the designated frontline healthcare professionals for hyperacute stroke assessments. For the present study, these stroke nurses also screened the patients with the V-FAST checklist as part of routine care; doctors were not involved in delivering V-FAST. The V-FAST screen for visual impairment is based on pre-set abnormality criteria, whilst the National Institutes of Health Stroke Scale evaluates stroke severity (Table 1). Where feasible, the order of assessment was randomised using a simple alternating sequence to minimise systematic bias. Overlapping visual components were assessed once but recorded separately. Some V-FAST sections required no additional testing, as it standardises visual field assessment, which is included in the National Institutes of Health Stroke Scale (Table 1).

**Table 1.** Comparison of elements included in V-FAST, national institutes of health stroke scale, and orthoptist assessments.

	Visual symptoms and observations	Eye position and movement	Reading ability	Visual fields	Visual extinction/ inattention	Visual acuity
V-FAST	✓	✓	✓	✓	Extinction only	X
National Institutes of Health Stroke Scale	X	Horizontal gaze only	X	Hemianopia only	Spatial neglect	X
Orthoptist assessment	✓	✓	✓	✓	✓	✓

V-FAST, Vision, Face-Arm-Speech-Time.

The V-FAST checklist was provided in print. Stroke nurses received a training package including instructions, a step-by-step video, and a self-training module (www.vision-research.co.uk) to ensure consistent application and interpretation across screeners.

Routine orthoptic evaluation was performed by a trained orthoptist and included a structured assessment of clinical history, visual acuity, ocular alignment, eye movements, visual fields, visual neglect, and visual perception. Visual acuity was assessed using standard bedside methods, ocular alignment and motility were evaluated using cover testing and pursuit/saccade assessment, and visual fields were assessed by confrontation. Neglect and perceptual deficits were evaluated using standard bedside screening techniques. Assessments were ideally conducted the same day as V-FAST to minimise recovery bias, though some delays occurred due to service availability. The orthoptic exam included all V-FAST domains and served as the reference standard for comparison (Table 1).

### Outcome and process evaluation

The primary outcome was the binary presence or absence of visual impairment on V-FAST in patients with suspected stroke, based on V-FAST detection of abnormal eye alignment or movement, impaired reading, visual field loss and extinction. Secondary outcomes included sensitivity, specificity, positive predictive value and negative predictive value of V-FAST compared with National Institutes of Health Stroke Scale and orthoptic assessments (reference standard). Each V-FAST domain was also evaluated individually against matching National Institutes of Health Stroke Scale and orthoptic items.

Screeners (stroke nurses) completed feedback surveys at the beginning and end of the study. Responses assessed clarity and usability of the checklist, perceived screening time, and satisfaction at baseline and follow-up (Supplemental Figures 2 and 3). Feedback was analysed descriptively using counts, percentage, and narrative summaries.

### Statistical analysis

Agreement between V-FAST, National Institutes of Health Stroke Scale and orthoptic assessments was evaluated using Cohen's Kappa (κ), with categories defined as poor (0.0–0.20), fair (0.21–0.40), moderate (0.41–0.60), substantial (0.61–0.80), and almost perfect (0.81–1.0).^7,8^ Sensitivity, specificity, positive predictive value and negative predictive value were calculated with 95% confidence intervals (CI). Sensitivity was defined as the proportion of patients with visual impairment correctly identified and specificity as the proportion without impairment correctly identified. Analyses were performed using StataCorp 2019 (Release 16).

Two pre-defined sub-group analyses were conducted: (1) V-FAST performance in FAST-positive vs. FAST-negative cases, and (2) the impact of timing between V-FAST and orthoptic assessments, particularly in discordant cases, to assess potential recovery effects.

### Sample size

The sample size calculation was based on estimating the sensitivity of V-FAST for detecting visual impairment compared with the orthoptic assessment (reference standard). A sensitivity of 93% was assumed from preliminary pre-hospital pilot data^5^ which is slightly lower than that reported for the Visual Impairment Screening Assessment tool (97%).^6^ Assuming a 60% prevalence of visual impairment in the suspected stroke population, a minimum of 150 screened patients was required to ensure that the lower bound of the 95% CI for V-FAST sensitivity would be ≥85%. To provide sufficient precision for comparison with the reference standard, at least 100 orthoptic assessments were required.^9^

## Results

The hospital provides stroke services for approximately 250,000 people, with around 1300 confirmed stroke admissions annually. Consecutive patients with suspected stroke presenting during routine clinical hours, who met the eligibility criteria were screened. A total of 282 adults with suspected stroke were assessed; 158 (56.0%) had confirmed stroke. Median age among stroke-confirmed patients was 74.5 years (IQR: 64–82), and 54.4% were males. V-FAST was completed in 98.1%, and National Institutes of Health Stroke Scale in 99.4% of stroke-confirmed patients. Orthoptic assessments were conducted in 103 patients, 83 of whom had confirmed stroke (Figure 1). Baseline characteristics are shown in Table 2 (confirmed stroke) and Supplemental Table 1 (entire suspected stroke cohort). This study cohort represents approximately 21% of the annual stroke cases seen at this hospital.

**Figure 1.**
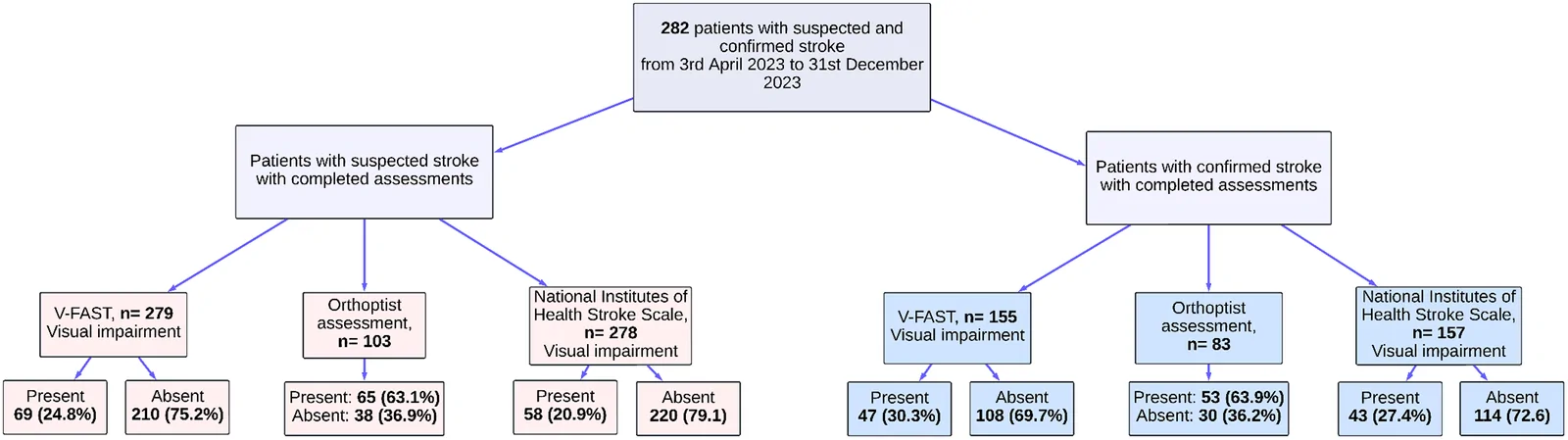
Flow diagram of consecutive patients with suspected and confirmed stroke assessed with V-FAST, national institutes of health stroke scale and orthoptist (standard reference) visual assessment.

V-FAST, Vision-Face-Arm-Speech-Time.

**Table 2.** Baseline demographic and clinical characteristics of patients with confirmed stroke.

Characteristic	N = 158
Age (years), median (IQR)	74.5 (64–82)
Male, n(%)	86 (54.4)
White, n(%)	144 (91.1)
**Type of residence, n(%)**	
Cohabiting	78 (49.4)
Lives alone	46 (29.1)
Lives in nursing home	6 (3.8)
Unknown	28 (17.7)
Body mass index, mean ± SD	26.7 ± 7.4
Systolic blood pressure (mmHg), mean ± SD	154 ± 34
Diastolic blood pressure (mmHg), mean ± SD	83 ± 73
**Previous medical history**	
Previous stroke, n(%)	19 (12)
Previous myocardial infarction, n(%)	14 (8.9)
Hypertension, n(%)	95 (60.1)
Diabetes, n(%)	39 (24.7)
Thrombolysis, n(%)	26 (16.5)
Thrombectomy, n(%)	7 (4.4)
Pre-stroke mRS score, median (IQR)	1 (0–2)
Discharge mRS score, median (IQR)	2 (1–4)
Barthel index score, median (IQR)	12 (6–18)
**Occlusion site, n(%)**	
Lacunar anterior circulation infarction	35 (22.6)
Partial anterior circulation infarction	29 (18.7)
Total anterior circulation infarction	17 (11.0)
Posterior circulation infarction	15 (9.7)
Transient ischaemic stroke	25 (13.5)
Primary intracerebral haemorrhage	22 (12.25)
Other	15 (12.25)

IQR, interquartile range; mRS, modified Rankin scale; SD, standard deviation.

### Visual impairment detection

In the confirmed stroke group, V-FAST detected visual impairment in 47/155 (30.3%) and National Institutes of Health Stroke Scale in 43/157 (27.4%) patients. Among those with orthoptic assessments, 53/83 (63.9%) had confirmed visual impairment. V-FAST identified 25/53 (47.2%) of these, while National Institutes of Health Stroke Scale identified 18/53 (34.0%). False positives were minimal (2/30; 6.6%) for both tools (Table 3). Analogous data for the suspected stroke group are shown in Supplemental Table 2.

**Table 3.** Detection of visual impairment by V-FAST, National Institutes of Health Stroke Scale, and orthoptic assessment in the confirmed stroke group.

Population	Total N	Assessment checklist	Visual impairment n (%)	Without visual impairment n (%)
**Without completed orthoptist assessment**	157	National Institutes of Health Stroke Scale*	43 (27.4)	114 (72.6)
	155	V-FAST*	47 (30.3)	108 (69.7)
	83	Orthoptist assessment	53 (63.9)	30 (36.2)
Completed orthoptist assessment with **presence** of visual impairment	53	National Institutes of Health Stroke Scale**	18 (34)	35 (66)
	53	V-FAST**	25 (47.2)	28 (52.8)
Completed orthoptist assessment with **absence** of visual impairment	30	National Institutes of Health Stroke Scale**	2 (6.6)	24 (93.4)
	30	V-FAST**	2 (6.6)	24 (93.4)

*Participants who did not receive an orthoptist assessment (and were not confirmed to have VI by orthoptic assessment), **participants who received an orthoptist assessment, and VI was confirmed by orthoptic assessment.

V-FAST showed higher detection of visual impairment than National Institutes of Health Stroke Scale in FAST-negative strokes (21.3% vs 16.3%). Among orthoptic-confirmed cases with FAST-negative stroke, V-FAST identified visual impairment in 24% of patients compared with 16.7% for National Institutes of Health Stroke Scale (Figure 2).

**Figure 2.**
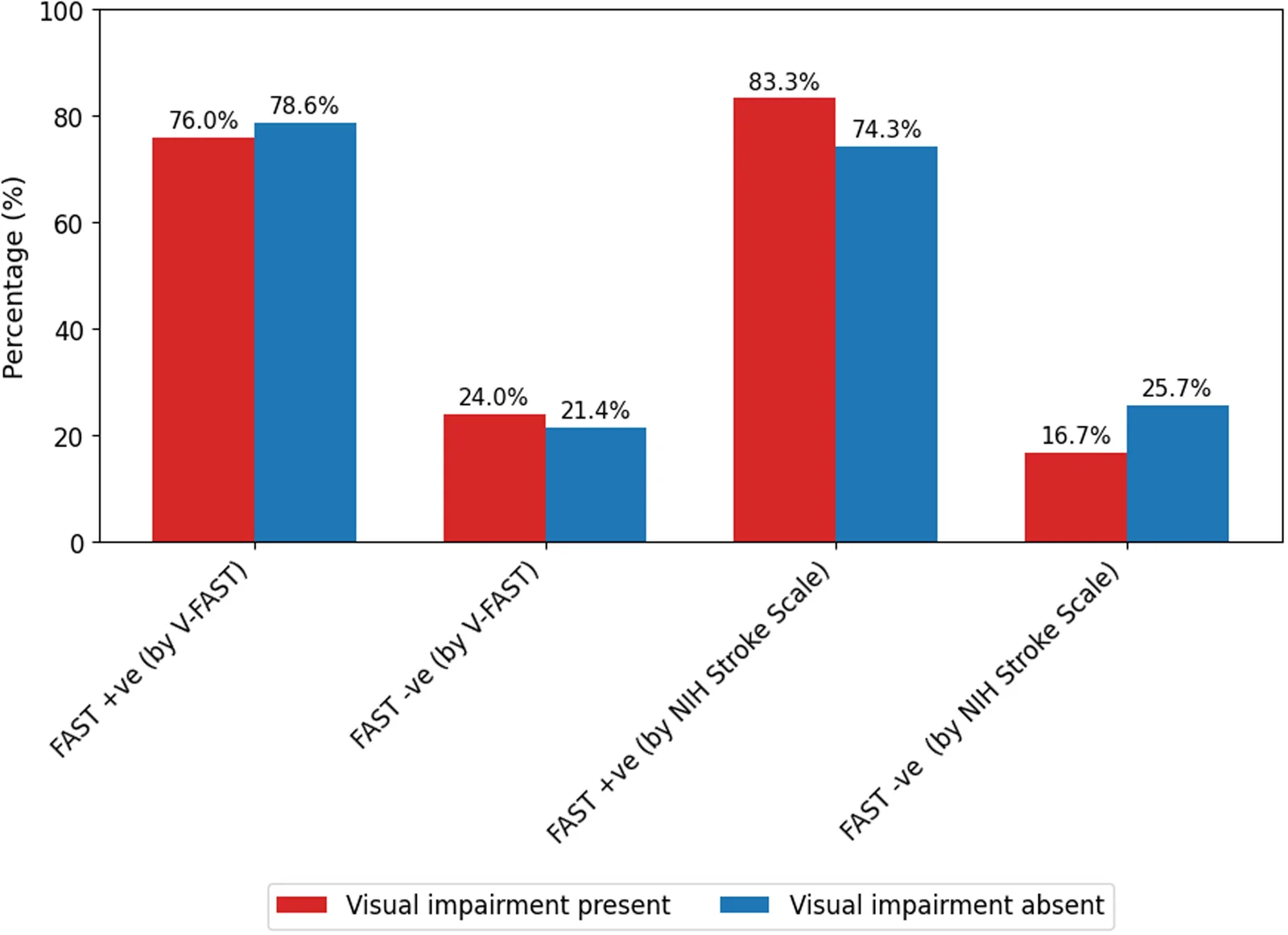
Distribution of confirmed stroke only with presence of visual impairment detected by orthoptist assessment stratified by FAST status (positive and negative) and by visual impairment status using V-FAST and national institutes of health stroke scale. Proportions represent the number of FAST-positive and FAST-negative individuals within the group identified as visually impaired and not visually impaired by each screening tool.

FAST, Face-Arm-Speech-Time.

### Agreement and diagnostic performance of v-fast compared with national institutes of health stroke scale and orthoptic assessment

Compared to National Institutes of Health Stroke Scale, V-FAST achieved sensitivity of 78.7%, specificity of 97.2%, positive predictive value of 92.5%, negative predictive value of 91.2%, and substantial agreement (κ=0.79, *p* < 0.001). The visual field sub-section had the strongest agreement (κ=0.79, *p* < 0.001). Visual fields had the highest sub-section agreement (κ=0.79, *p* < 0.001), while the eye movement sub-section showed lower agreement (κ=0.65) (Table 4). Results for suspected and non-stroke groups are in Supplemental Tables 3 and 4, respectively.

**Table 4.** Performance and agreement of visual impairment detection of V-FAST versus national institutes of health stroke scale in those with confirmed stroke.

National institutes of health stroke scale	V-FAST						
	Visual impairment			Diagnostic measure*		(V-FAST vs National Institutes of Health Stroke Scale)	
	Total	Present n (%)	Absent n (%)	Sensitivity (95% CI)	Specificity (95% CI)	*Kappa coefficient*	*P* value
				Positive predictive value (95% CI)	Negative predictive value (95% CI)		
Overall † n = 154							
Any defect present	40	37 (24)	3 (1.9)	78.7 (64.3–89.3)	97.2 (92–99.4)	0.79	**<0.001**
No defect on National Institutes of Health Stroke Scale	114	10 (6.5)	104 (67.5)	92.5 (79.6–98.4)	91.2 (84.5–95.7)		
Eye movements defect ‡ n = 150							
Present	27	19 (12.7)	8 (5.3)	73.1 (52.2–88.4)	93.5 (87.7–97.2)	0.65	**<0.001**
Absent	123	7 (4.7)	116 (77.3)	70.4 (49.8–86.2)	94.3 (88.6–97.7)		
Visual fields defect § n = 152							
Present	40	31 (20.4)	9 (5.9)	91.2 (76.3–98.1)	92.4 (86–96.5)	0.79	**<0.001**
Absent	112	3 (2)	109 (71.7)	77.5 (61.5–89.2)	97.3 (92.4–99.4)		
Visual inattention/extinction defect || n = 152							
Present	27	22 (14.5)	5 (3.3)	66.7 (48.2–82)	95.8 (90.5–98.6)	0.67	**<0.001**
Absent	125	11 (7.2)	114 (75)	81.5 (61.9–93.7)	91.2 (84.8–95.5)		

*V-FAST against National Institutes of Health Stroke Scale; National Institutes of Health Stroke Scale is the reference standard. † Defined as any visual defect in any section of National Institutes of Health Stroke Scale and any visual defect in any section of the V-FAST, ‡ Defined as any visual defect in eye movement section of National Institutes of Health Stroke Scale and any visual defect in eye movement section of the V-FAST, § Defined as any visual defect in visual fields section of National Institutes of Health Stroke Scale and any visual defect in visual fields section of the V-FAST, || Defined as any visual defect in visual inattention/extinction section of National Institutes of Health Stroke Scale and any visual defect in visual inattention/extinction section of the V-FAST. The observation and reading sections in V-FAST are not available in the National Institutes of Health Stroke Scale, so comparison was not performed for these sections. Bold fonts indicate statistical significance.

Compared to orthoptic assessment, V-FAST had sensitivity 92.6%, specificity 50.0%, positive predictive value 47.2%, and negative predictive value 93.3%, with fair agreement overall (κ=0.34, *p* = 0.0001) (Table 5). Visual inattention had the highest subsection agreement (κ=0.67), while eye movement remained weakest (κ=0.14) (Table 5). Results for suspected and non-stroke groups are in Supplemental Tables 5 and 6.

**Table 5.** Performance and agreement of visual impairment detection of V-FAST versus orthoptic assessment in those with confirmed stroke.

Orthoptic	V-FAST						
	Visual impairment			Diagnostic measure*		(V-FAST vs orthoptic)	
	Total	Present n (%)	Absent n (%)	Sensitivity (95% CI)	Specificity (95% CI)	*Kappa coefficient*	*P* value
				Positive predictive value (95% CI)	Negative predictive value (95% CI)		
Overall † n = 83							
Any defect present	53	25 (30.1)	28 (33.7)	92.6 (75.7–99.1)	50 (36.3–63.7)	0.34	**0.0001**
No defect on orthoptic	30	2 (2.4)	28 (33.7)	47.2 (33.3–61.4)	93.3 (78–99.2)		
Eye movements defect § n = 81							
Present	34	8 (9.9)	26 (32.1)	61.5 (31.6–86.1)	61.8 (49.2–73.3)	0.14	0.06
Absent	47	5 (6.2)	42 (51.9)	23.5 (10.7–41.2)	89.4 (77–96.5)		
Visual fields defect || n = 78							
Present	23	13 (16.7)	10 (12.8)	72.2 (46.5–90.3)	83.3 (71.5–91.7)	0.50	**<0.001**
Absent	55	5 (6.4)	50 (64.1)	56.5 (34.5–76.8)	91 (80–97)		
Visual inattention/extinction defect # n = 79							
Present	18	13 (16.5)	5 (6.3)	76.5 (50.1–93.2)	92 (82.2–97.3)	0.67	**<0.001**
Absent	61	4 (5.1)	57 (72.2)	72.2 (46.5–90.3)	93.4 (84.1–98)		
Reading defect ** n = 72							
Present	14	10 (13.9)	4 (5.6)	71.4 (42–91.6)	93.1 (83.3–98.1)	0.64	**<0.001**
Absent	58	4 (5.6)	54 (75)	71.4 (42–90.3)	93.1 (83.3–98.1)		

*V-FAST against orthoptic assessment; orthoptic assessment is the reference standard. † Defined as any visual defect in any section of orthoptic assessment and any visual defect in any section of the V-FAST, § Defined as any visual defect in eye movement section of orthoptic assessment and any visual defect in eye movement section of the V-FAST, || Defined as any visual defect in visual fields section of orthoptic assessment and any visual defect in visual fields section of the V-FAST, # Defined as any visual defect in visual inattention/extinction section of orthoptic assessment and any visual defect in visual inattention/extinction section of the V-FAST, ** Defined as any visual defect in reading section of orthoptic assessment and any visual defect in reading section of the V-FAST. Bold fonts indicate statistical significance.

### Timing and discordant results

Among stroke patients assessed the same day (*n* = 36/83; 43.4%), V-FAST had fair agreement with orthoptic assessment. Sensitivity was 90.0%, specificity 53.8%, positive predictive value 43.0%, and negative predictive value 93.3%, and κ=0.33 (*p* = 0.008). Similarly, 47 (56.6%) were assessed on different days with sensitivity 94.1%, specificity 46.7%, positive predictive value 50.0%, negative predictive value 93.3%, and fair agreement (κ=0.34, *p* = 0.002). Same day assessments showed higher performance and agreement in visual fields, visual inattention/extinction and reading, while eye movement had poor performance and agreement (Supplemental Table 8). More assessments were performed on different days between V-FAST and orthoptic assessment than the same day in the suspected stroke and non-stroke groups (Supplemental Tables 7 and 9).

For V-FAST abnormal results, discrepancies were found in 21/83 (25.3%) cases where V-FAST detected abnormalities not confirmed by orthoptists, mostly involving visual field or eye movement (Supplemental Tables 10 and 11). Most discrepancies (13/21; 61.9%) occurred when assessments were on different days, suggesting fluctuating or transient symptoms.

For V-FAST normal results, in 44/83 (53.0%) cases, orthoptic assessments detected impairments not captured by V-FAST, particularly ocular motility deficits (Supplemental Tables 12 and 13). Most frequent discrepancies involved combined or subtle impairments.

#### Screener feedback

Sixteen nurses completed feedback at baseline and follow-up. At baseline, most found the instructions clear (*n* = 12, 75.0%), with three suggesting amendments for visual neglect, visual fields and reading (one each). The majority did not find the tests difficult (*n* = 14, 87.5%), and the median completion time was 5 min (range 2–10). No suggestions were made for additional instructions, rewording, or less information. Comments indicated uncertainty about the target patient group but suggested V-FAST could be useful in emergency department, general practice surgeries, or pre-hospital settings, particularly for ambulance staff.

At follow-up, among respondents, a median completion time of 5 min was reported and most performed the screening more than twice weekly. However, not all respondents answered every question. Of those who responded, two (40%) found the instructions clear from baseline; three (60%) did not. Despite this, none suggested amendments to the instructions or rewording, additional tests to add (*n* = 5), nor did any report challenges perform the tests (*n* = 5). While one nurse (25%) reported quicker completion over time, three (75%) found it look longer. One participant noted that, despite being a one-page form, the V-FAST added considerable time to the National Institutes of Health Stroke Scale assessment.

## Discussion

The V-FAST checklist was developed to detect common visual impairments in patients with suspected stroke during pre-hospital assessment, drawing from two large prospective stroke-vision studies.^1,10^ In this study, V-FAST demonstrated good sensitivity and specificity, compared to the National Institutes of Health Stroke Scale, with the strongest performance observed in patients with confirmed strokes. Agreement with the National Institutes of Health Stroke Scale was highest for visual inattention and field deficits but lowest for eye movement abnormalities, consistent with its design as a rapid tool for detecting prominent visual dysfunction. When compared with orthoptic assessments, V-FAST showed fair overall agreement, with substantial agreement for inattention, moderate agreement for visual field, and slight agreement for eye movement abnormalities. These findings support its utility in early identification of visual in hyperacute stroke admission settings beyond what is achieved by the National Institutes of Health Stroke Scale alone.

This study extends previous work by showing that V-FAST performs more effectively in the emergency department than earlier pre-hospital evaluations.^5^ The good sensitivity and higher specificity observed in the present study likely reflects the more controlled clinical environment and reduced time pressures in the emergency department, as well as the involvement of trained stroke nurses. Although the same educational package was used across studies,^5^ consistent and focused application within the emergency department may have contributed to improved accuracy.

As expected for a brief screening tool, V-FAST did not detect all impairments identified by orthoptics. Orthoptic assessments remain the gold-standard for identifying subtle or multiple visual impairments that are not feasible in emergency care settings. Nonetheless, V-FAST identified a broader range of impairments than the National Institutes of Health Stroke Scale, consistent with previous studies.^5,6,11^ This reflects its wider scope, which includes assessment of eye movements and reading alongside visual fields and inattention, addressing known limitations of the National Institutes of Health Stroke Scale, which includes only three vision-related items (horizontal eye movements, visual fields and inattention). This broader coverage is particularly relevant for identifying posterior circulation strokes, which frequently present with visual deficits but lack FAST-positive symptoms.^12^

V-FAST performed less effectively in non-stroke patients, suggesting its utility lies within hyperacute stroke pathways where visual dysfunction is more likely to be stroke-related. Some impairments identified in the non-stroke group may reflect co-existing ocular or neurological conditions, especially as only a minority of these patients received orthoptic assessment. Importantly, V-FAST identified more visual impairments than the National Institutes of Health Stroke Scale among FAST-negative stroke patients, supporting its potential role in improving recognition of posterior circulation strokes. Among patients who underwent orthoptic assessment and were confirmed to have visual impairments, the V-FAST checklist detected more abnormalities than the National Institutes of Health Stroke Scale, indicating added clinical value during early triage and addressing an important gap in current stroke care pathways.^12^

Agreement with orthoptic assessment varied by visual domain. Substantial agreement was observed for inattention, while agreement for visual fields was moderate, with only slight agreement for eye movements across patient groups. Reading assessments showed moderate agreement in suspected stroke patients and substantial agreement in confirmed stroke patients, reflecting the greater prominence of deficits in the latter group. These domain-level patterns are consistent with previous work using more detailed tools such as the VISA checklist,^6,11^ which also report stronger performance for inattention and visual field loss than for eye movement abnormalities. Together, these findings highlight the strengths of V-FAST in identifying clear visual dysfunction, while illustrating the limitations of rapid screening tools in detecting more subtle abnormalities.

Differences between V-FAST and orthoptic assessment may also reflect the timing of the evaluations. The V-FAST screening checklist was performed at emergency department presentation, while orthoptic assessments occurred between 2 h and 7 days later. Visual function can improve early after stroke and partial recovery during this interval may account for some apparent false positives on V-FAST. Such temporal effects highlight the challenge of comparing acute screening with later specialist assessment and are consistent with previous large-scale vision–stroke studies demonstrating that early visual recovery most prominent within the first month post-stroke.^13^

This study has several strengths, including a prospectively recruited cohort drawn from routine emergency department practice, screening by trained stroke nurses using a standardised educational package, and use of certified orthoptists as the reference standard. Although the cohort represents approximately 21% of annual stroke presentations at the study site, this reflects the pragmatic nature of real-world implementation. Patients who were unable to cooperate fully (e.g., due to aphasia, confusion, or severe impairment) were assessed in line with National Institutes of Health Stroke Scale guidelines to ensure inclusion while appropriately accounting for these limitations. However, several limitations must be acknowledged. This was a single-centre evaluation, which may limit generalisability. Screening was conducted when trained staff was available, and some patients who were rapidly transferred or presented outside the stroke pathway were not screened, reflecting pragmatic workflow constraints. Screening duration was not recorded, limiting understanding of time burden, and screener feedback was collected only via surveys, providing limited insight into usability.

Differences in the numbers of patients assessed by V-FAST/National Institutes of Health Stroke Scale and orthoptic examination may have influenced these estimates. Assessments were conducted independently as part of routine clinical care, with stroke nurses and orthoptists blinded to each other's findings at the time of assessment. V-FAST was typically performed prior to orthoptic assessment without randomisation, which may introduce an order effect. Assessments were conducted by multiple clinicians, reflecting usual emergency department workflow; however, this may introduce inter-rater variability, while reliance on a limited number of orthoptists may also introduce observer bias.

The V-FAST checklist improves detection of stroke-related visual impairments in an emergency department setting compared to the National Institutes of Health Stroke Scale and may aid identification of FAST-negative and posterior circulation strokes that might otherwise be missed. However, it is not a substitute for comprehensive specialist evaluation and does not meet the full core outcome set recommended for vision screening in acute stroke units and is not validated for use as a vision screening check in acute stroke units. V-FAST is best considered an adjunctive tool that supports early recognition of visual deficits and facilitates timely referral for detailed assessment. Future multi-centre studies should further evaluate its performance and define its optimal integration into stroke care pathways. V-FAST may provide emergency clinicians with a simple, structured approach to augment the National Institutes of Health Stroke Scale and improve early recognition of visual impairment, particularly in patients with suspected posterior or FAST-negative stroke.

**Clinical messages**

- Vision-face-arm-speech-time (V-FAST) improves early detection of visual impairment in patients with suspected stroke, particularly in FAST-negative strokes, where it outperforms the National Institutes of Health Stroke Scale in emergency department settings.
- V-FAST is particularly useful for identifying visual deficits in FAST-negative strokes.
- Rapid V-FAST screening can be feasibly delivered by trained emergency department stroke nurses within routine workflows.
- V-FAST should complement, but not replace, comprehensive orthoptic assessment and formal vision screening in acute stroke care.

## Supplemental Material

sj-docx-1-cre-10.1177_02692155261450137 - Supplemental material for Evaluating the use of vision-face-arm-speech-time in stroke admissions: A comparative study with national institutes of health stroke scale and orthoptist assessment in an emergency department Supplemental material, sj-docx-1-cre-10.1177_02692155261450137 for Evaluating the use of vision-face-arm-speech-time in stroke admissions: A comparative study with national institutes of health stroke scale and orthoptist assessment in an emergency department by Muath Alobaida, Deirdre A. Lane, Stephanie L. Harrison, Gregory Y. H. Lip and Fiona J. Rowe in Clinical Rehabilitation

## Data Availability

The entire deidentified dataset, data dictionary and analytic code for this investigation are available upon request, from the date of article publication by contacting Fiona Rowe, PhD, at email rowef@liverpool.ac.uk.
