# Supplementary material for: Evaluating the use of vision-face-arm-speech-time in stroke admissions: A comparative study with national institutes of health stroke scale and orthoptist assessment in an emergency department
Source: Clin Rehabil. 2026 May 14;40(11):1494–505. doi: 10.1177/02692155261450137 (PMC13736282; doi:10.1177/02692155261450137)
Supplement: Supplementary file 1 [file sj-docx-1-cre-10.1177_02692155261450137.docx]

**Supplementary Figure 1.** A flow chart shows the Vision-Face-Arm-Speech and Time (V-FAST) study design.

**Supplementary Figure 2** Baseline feedback assessment form.

**Supplementary Figure 3** Follow-up feedback assessment form.

**Supplementary Figure 3.** Follow-up feedback assessment form (continued)

**Supplementary Table 1.** Baseline demographic and clinical characteristics of people who presented with suspected stroke (entire population)

| **Characteristic** | **Value**  **(N= 282)** |
| --- | --- |
|  |  |
| Age (years), median (IQR) | 73 (62–82) |
| Male, n (%) | 137 (48.6) |
| White, n (%) | 262 (93.2) |
| **Type of residence, n (%)**  Cohabiting  Lives alone  Lives in nursing home  Unknown | 133 (47.0)  69 (24.5)  15 (5.5)  65 (23.0) |
| Body mass index, mean ± SD | 27.6 ± 6.99 |
| Systolic blood pressure (mmHg), mean ± SD | 148 ± 34 |
| Diastolic blood pressure (mmHg), mean ± SD | 82 ± 21 |
| **Previous medical history** |  |
| Previous stroke, n (%) | 48 (17.2) |
| Myocardial infarction, n (%) | 21 (7.5) |
| Hypertension, n (%) | 146 (51.7) |
| Diabetes, n (%) | 68 (24.1) |
| Thrombolysis, n (%) | 27 (9.6) |
| Thrombectomy, n (%) | 7 (2.5) |
| Pre-stroke mRS score, median (IQR) | 1 (0–2) |
| Discharge mRS score, median (IQR) | 1 (0–3) |
| Barthel index score, median (IQR) | 13 (7–20) |
| **Confirmed stroke, n (%)** |  |
| No | 123 (44) |
| Yes | 158 (56) |

IQR, interquartile range; mRS, modified Rankin scale; standard deviation (SD).

**Supplementary Table 2.** Detection of visual impairment by V-FAST, National Institutes of Health Stroke Scale, and orthoptic assessment in the **suspected stroke** population and confirmed stroke group.

| **Population** | **Assessment checklist** | **N with visual impairment** | **N without visual impairment** | **Total N** |
| --- | --- | --- | --- | --- |
| **Overall population** | National Institutes of Health Stroke Scale* | 58 (20.9) | 220 (79.1) | 278 |
|  | V-FAST* | 69 (24.8) | 210 (75.2) | 279 |
|  | Orthoptist assessment | 65 (63.1) | 38 (36.9) | 103 |
|  | | | | |
| **Confirmed stroke** | National Institutes of Health Stroke Scale* | 43 (27.4) | 114 (72.6) | 157 |
|  | V-FAST* | 47 (30.3) | 108 (69.7) | 155 |
|  | Orthoptist assessment | 53 (63.9) | 30 (36.2) | 83 |
| **Completed orthoptist assessment with visual impairment** | | | | |
| **Confirmed stroke** | National Institutes of Health Stroke Scale** | 18 (34%) | 35 (66%) | 53 |
|  | V-FAST** | 25 (47.2) | 28 (52.8) | 53 |
| **Completed orthoptist assessment with absence of visual impairment** | | | | |
| **Confirmed stroke** | National Institutes of Health Stroke Scale** | 2 (6.6) | 24 (93.4) | 30 |
|  | V-FAST** | 2 (6.6) | 24 (93.4) | 30 |

* Participants who did not receive an orthoptist assessment (and were not confirmed to have visual impairment by orthoptic assessment), ** participants who received an orthoptist assessment, and visual impairment was confirmed by orthoptic assessment.

**Supplementary Table 3.** Performance and agreement of visual impairment detection of V-FAST versus National Institutes of Health Stroke Scale in **suspected stroke** population.

| V-FAST  National  Institutes of  Health Stroke  Scale | Visual impairment | | | Diagnostic measure * | | (V-FAST vs National Institutes of Health Stroke Scale) | |
| --- | --- | --- | --- | --- | --- | --- | --- |
|  | **Total** | **Present**  **N (%)** | **Absent**  **N (%)** | **Sensitivity (95% CI)** | **Specificity (95% CI)** | ***Kappa coefficient*** | ***P* value** |
|  |  |  |  | **Positive predictive value (95% CI)** | **Negative predictive value (95% CI)** |  |  |
| Overall † n= 275 | | | | | | | |
| Any defect present | 55 | 46 (16.7) | 9 (3.3) | 67.6 (55.2–78.5) | 95.7 (92–98) | 0.68 | **<0.001** |
| No defect on National Institutes of Health Stroke Scale | 220 | 22 (8) | 198 (72) | 83.6 (71.2–92.2) | 90 (85.3–93.6) |  |  |
| Eye movements defect ‡ n= 269 | | | | | | | |
| Present | 32 | 22 (8.2) | 10 (3.7) | 59.5 (42.1–75.2) | 95.7 (92.2–98) | 0.58 | **<0.001** |
| Absent | 237 | 15 (5.6) | 222 (82.5) | 68.8 (50–84) | 93.7 (89.8–96.4) |  |  |
| Visual fields defect § n= 271 | | | | | | | |
| Present | 48 | 34 (12.5) | 14 (5.2) | 81 (66–91.4) | 94 (90–96.6) | 0.71 | **<0.001** |
| Absent | 223 | 8 (3) | 215 (79.3) | 70.8 (56–83) | 96.4 (93.1–98.4) |  |  |
| Visual inattention/extinction defect ‖ n= 271 | | | | | | | |
| Present | 29 | 23 (8.5) | 6 (2.2) | 64 (46.2–79.2) | 97.4 (94.5–99.1) | 0.67 | **<0.001** |
| Absent | 242 | 13 (4.8) | 229 (84.5) | 79.3 (60.3–92) | 94.6 (91–97.1) |  |  |

* V-FAST against National Institutes of Health Stroke Scale; National Institutes of Health Stroke Scale is the reference standard. † Defined as any visual defect in any section of National Institutes of Health Stroke Scale and any visual defect in any section of the V-FAST, ‡ Defined as any visual defect in eye movement section of National Institutes of Health Stroke Scale and any visual defect in eye movement section of the V-FAST, § Defined as any visual defect in visual fields section of National Institutes of Health Stroke Scale and any visual defect in visual fields section of the V-FAST, ‖ Defined as any visual defect in visual inattention/extinction section of National Institutes of Health Stroke Scale and any visual defect in visual inattention/extinction section of the V-FAST. The observation and reading sections in V-FAST are not available in the National Institutes of Health Stroke Scale, so comparison was not performed for these sections. Bold fonts indicate statistical significance.

**Supplementary Table 4.** Performance and agreement of visual impairment detection of V-FAST versus National Institutes of Health Stroke Scale in the **non-stroke** population.

| V-FAST  National  Institutes of Health  Stroke Scale | Visual impairment | | | Diagnostic measure * | | (V-FAST vs National Institutes of Health Stroke Scale) | | |
| --- | --- | --- | --- | --- | --- | --- | --- | --- |
|  | **Total** | **Present n (%)** | **Absent**  **n (%)** | **Sensitivity (95% CI)** | **Specificity (95% CI)** | ***Kappa coefficient*** | ***P* value** | |
|  |  |  |  | **Positive predictive value (95% CI)** | **Negative predictive value (95% CI)** |  |  |  |
| Overall † n=121 | | | | | | | | |
| Any defect present | 15 | 9 (7.4) | 6 (5) | 43 (22–66) | 94 (87.4–98) | 0.42 | **<0.001** | |
| No defect on National Institutes of Health Stroke Scale | 106 | 12 (9.9) | 94 (77.7) | 60 (32.3–83.7) | 89 (81.1–94) |  |  |  |
| Eye movements defect ‡ n=119 | | | | | | | | |
| Present | 5 | 3 (2.5) | 2 (1.7) | 27.3 (6–61) | 98.1 (93.5–99.8) | 0.34 | **<0.001** | |
| Absent | 114 | 8 (6.7) | 106 (89.1) | 60 (14.7–94.7) | 93 (86.6–96.9) |  |  |  |
| Visual fields defect § n=119 | | | | | | | |  |
| Present | 8 | 3 (2.5) | 5 (4.2) | 37.5 (8.5–75.5) | 95.5 (90–98.5) | 0.33 | **0.0002** | |
| Absent | 111 | 5 (4.2) | 106 (89.1) | 37.5 (8.5–75.5) | 95.5 (90–98.5) |  |  |  |
| Visual inattention/extinction defect ‖ n=119 | | | | | | | | |
| Present | 2 | 1 (0.8) | 1 (0.8) | 33.3 (0.8–90.6) | 99.1 (95.3–100) | 0.39 | **<0.001** | |
| Absent | 117 | 2 (1.7) | 115 (96.6) | 50 (1.3–98.7) | 98.3 (94–99.8) |  |  |  |

* V-FAST against National Institutes of Health Stroke Scale; National Institutes of Health Stroke Scale is the reference standard. † Defined as any visual defect in any section of National Institutes of Health Stroke Scale and any visual defect in any section of the V-FAST, ‡ Defined as any visual defect in eye movement section of National Institutes of Health Stroke Scale and any visual defect in eye movement section of the V-FAST, § Defined as any visual defect in visual fields section of National Institutes of Health Stroke Scale and any visual defect in visual fields section of the V-FAST, ‖ Defined as any visual defect in visual inattention/extinction section of National Institutes of Health Stroke Scale and any visual defect in visual inattention/extinction section of the V-FAST. The observation and reading sections in V-FAST are not available in the National Institutes of Health Stroke Scale, so comparison was not performed for these sections. Bold fonts indicate statistical significance.

**Supplementary Table 5.** Performance and agreement of visual impairment detection of V-FAST versus orthoptic assessment in the **suspected stroke** population.

| V-FAST  Orthoptic | Visual impairment | | | Diagnostic measure * | | (V-FAST vs orthoptic) | |
| --- | --- | --- | --- | --- | --- | --- | --- |
|  | **Total** | **Present n (%)** | **Absent**  **n (%)** | **Sensitivity (95% CI)** | **Specificity (95% CI)** | ***Kappa coefficient*** | ***P* value** |
|  |  |  |  | **Positive predictive value (95% CI)** | **Negative predictive value (95% CI)** |  |  |
| Overall † n=103 | | | | | | | |
| Any defect present ‡ | 65 | 33 (32) | 32 (31.1) | 91.7 (77.5–98.2) | 52.2 (39.7–64.6) | 0.37 | **<0.001** |
| No defect on orthoptic | 38 | 3 (2.9) | 35 (34) | 50.8 (38.1–63.4) | 92.1 (78.6–98.3) |  |  |
| Eye movements defect § n=100 | | | | | | | |
| Present | 43 | 11 (11) | 32 (32) | 61.1 (35.7–82.7) | 61 (49.6–71.6) | 0.14 | **0.04** |
| Absent | 57 | 7 (7) | 50 (50) | 25.6 (13.5–41.2) | 87.7 (76.3–95) |  |  |
| Visual fields defect ‖ n=96 | | | | | | | |
| Present | 26 | 14 (14.6) | 12 (12.5) | 66.7 (43–85.4) | 84 (73.7–91.4) | 0.47 | **<0.001** |
| Absent | 70 | 7 (7.3) | 63 (65.6) | 53.8 (33.4–73.4) | 90 (80.5–96) |  |  |
| Visual inattention/extinction defect # n=96 | | | | | | | |
| Present | 19 | 14 (14.6) | 5 (5.2) | 77.8 (52.4–93.6) | 93.6 (85.7–98) | 0.70 | **<0.001** |
| Absent | 77 | 4 (4.2) | 73 (76) | 73.7 (49–91) | 95 (87.2–98.6) |  |  |
| Reading defect ** n=90 | | | | | | | |
| Present | 14 | 10 (11.1) | 4 (4.4) | 59 (33–81.6) | 94.5 (86.6–98.5) | 0.57 | **<0.001** |
| Absent | 76 | 7 (7.8) | 69 (76.7) | 71.4 (42–91.6) | 90.8 (82–96.2) |  |  |

* V-FAST against orthoptic assessment; orthoptic assessment is the reference standard. † Defined as any visual defect in any section of orthoptic assessment and any visual defect in any section of the V-FAST, ‡ 2 missing, § Defined as any visual defect in eye movement section of orthoptic assessment and any visual defect in eye movement section of the V-FAST, ‖ Defined as any visual defect in visual fields section of orthoptic assessment and any visual defect in visual fields section of the V-FAST, # Defined as any visual defect in visual inattention/extinction section of orthoptic assessment and any visual defect in visual inattention/extinction section of the V-FAST, ** Defined as any visual defect in reading section of orthoptic assessment and any visual defect in reading section of the V-FAST. Bold fonts indicate statistical significance.

**Supplementary Table 6.** Performance of visual impairment detection of V-FAST versus orthoptic assessment in the **non-stroke** status.

| V-FAST  Orthoptic | Visual impairment | | | | Diagnostic measure * | | | (V-FAST vs orthoptic) | |
| --- | --- | --- | --- | --- | --- | --- | --- | --- | --- |
|  | **Total** | **Present n (%)** | **Absent n (%)** | **Sensitivity (95% CI)** | | | **Specificity (95% CI)** | ***Kappa coefficient*** | ***P* value** |
|  |  |  |  | **Positive Predictive value (95% CI)** | | | **Negative Predictive value (95% CI)** |  |  |
| Overall † n=20 | | | | | | | | | |
| Any defect present | 12 | 8 (40) | 4 (20) | 89 (51.8–99.7) | | | 63.6 (30.8–89.1) | 0.51 | **0.0085** |
| No defect on orthoptic | 8 | 1 (5) | 7 (35) | 66.7 (35–90.1) | | | 87.5 (47.3–99.7) |  |  |
| Eye movements defect § n=19 | | | | | | | | | |
| Present | 9 | 3 (15.8) | 6 (31.6) | 60 (14.7–94.7) | | | 57.1 (29–82.3) | 0.14 | 0.26 |
| Absent | 10 | 2 (10.5) | 8 (42.1) | 33.3 (7.5–70.1) | | | 80 (44.4–97.5) |  |  |
| Visual fields defect ‖ n=18 | | | | | |  | | | |
| Present | 3 | 1 (5.6) | 2 (11.1) | 33.3 (0.8–90.6) | | | 86.7 (59.5–98.3) | 0.20 | 0.19 |
| Absent | 15 | 2 (11.1) | 13 (72.2) | 33.3 (0.8–90.6) | | | 86.7 (59.5–98.3) |  |  |
| Visual inattention/extinction defect # n=19 | | | | | | | | | |
| Present | 0 | 0 | 0 | - | | | - | - | - |
| Absent | 19 | 1 (5.3) | 18 (94.7) | - | | | - |  |  |
| Reading defect ** n=18 | | | | | | | | | |
| Present | 1 | 1 (5.9) | 0 | 100 (2.5–100) | | | 100 (79.4–100) | 1 | **<0.001** |
| Absent | 16 | 0 | 16 (94.1) | 100 (2.5–100) | | | 100 (79.4–100) |  |  |

* V-FAST against orthoptic assessment; orthoptic assessment is the reference standard. † Defined as any visual defect in any section of orthoptic assessment and any visual defect in any section of the V-FAST, § Defined as any visual defect in eye movement section of orthoptic assessment and any visual defect in eye movement section of the V-FAST, ‖ Defined as any visual defect in visual fields section of orthoptic assessment and any visual defect in visual fields section of the V-FAST, # Defined as any visual defect in visual inattention/extinction section of orthoptic assessment and any visual defect in visual inattention/extinction section of the V-FAST, ** Defined as any visual defect in reading section of orthoptic assessment and any visual defect in reading section of the V-FAST. Bold fonts indicate statistical significance.

**Supplementary Table 7.** Subgroup analysis of performance and agreement on visual impairment detection of V-FAST versus orthoptic assessment in **suspected stroke** population stratified by assessments undertaken on the same or different dates.

| V-FAST  Orthoptic | Visual impairment | | | Diagnostic measure * | | (V-FAST vs orthoptic) | | |
| --- | --- | --- | --- | --- | --- | --- | --- | --- |
|  | **Total** | **Present**  **n (%)** | **Absent**  **n (%)** | **Sensitivity (95% CI)** | **Specificity (95% CI)** | ***Kappa coefficient*** | ***P* value** | |
|  |  |  |  | **Positive predictive value (95% CI)** | **Negative predictive value (95% CI)** |  |  |  |
| Overall † n=103 | | | | | | | | |
| Any defect present ‡ | 65 | 33 (32) | 32 (31.1) | 91.7 (77.5–98.2) | 52.2 (39.7–64.6) | 0.37 | **<0.001** | |
| No defect on orthoptic | 38 | 3 (2.9) | 35 (33.9) | 50.8 (38.1–63.4) | 92.1 (78.6–98.3) |  |  |  |
| Overall † Same day n=45 | | | | | | | | |
| Any defect present | 25 | 11 (24.5) | 14 (31.1) | 84.6 (54.6–98.1) | 56.3 (37.7–73.6) | 0.32 | **0.006** | |
| No defect on orthoptic | 20 | 2 (4.4) | 18 (40) | 44 (24.4–65.1) | 90 (68.3–99) |  |  |  |
| Overall † Different day n= 58 | | | | | | | | |
| Any defect present | 40 | 22 (37.9) | 18 (31) | 95.7 (78.1–99.9) | 48.6 (31.4–66) | 0.39 | **0.0002** | |
| No defect on orthoptic | 18 | 1 (1.7) | 17 (29.3) | 55 (38.5–71) | 94.4 (73–100) |  |  |  |
| Eye movements defect § n= 100 | | | | | | | | |
| Present | 43 | 11 (11) | 32 (32) | 61.1 (35.7–82.7) | 61 (49.6–71.6) | 0.14 | **0.04** | |
| Absent | 57 | 7 (7) | 50 (50) | 25.6 (13.5–41.2) | 87.7 (76.3–95) |  |  |  |
| Eye movements defect § Same day n= 45 | | | | | | | | |
| Present | 17 | 2 (4.4) | 15 (33.3) | 50 (6.8–93.2) | 63.4 (47–78) | 0.05 | 0.29 | |
| Absent | 28 | 2 (4.4) | 26 (57.8) | 11.8 (1.5–36.4) | 93 (76.5–99.1) |  |  |  |
| Eye movements defect § Different day n= 55 | | | | | | | | |
| Present | 26 | 9 (16.4) | 17 (30.9) | 64.3 (35.1–87.2) | 58.5 (42.1–73.7) | 0.18 | 0.07 | |
| Absent | 29 | 5 (9.1) | 24 (43.6) | 34.6 (17.2–55.7) | 83 (64.2–94.2) |  |  |  |
| Visual fields defect ‖ n=96 | | | | | | | | |
| Present | 26 | 14 (14.6) | 12 (12.5) | 66.7 (43–85.4) | 84 (73.7–91.4) | 0.47 | **<0.001** | |
| Absent | 70 | 7 (7.3) | 63 (65.6) | 54 (33.4–73.4) | 90 (80.5–96) |  |  |  |
| Visual fields defect ‖ Same day n=44 | | | | | | | | |
| Present | 11 | 6 (13.6) | 5 (11.4) | 75 (35–97) | 86.1 (70.5–95.3) | 0.53 | **0.0002** | |
| Absent | 33 | 2 (4.5) | 31 (70.5) | 54.5 (23.4–83.3) | 94 (80–99.3) |  |  |  |
| Visual fields defect ‖ Different day n=52 | | | | | | | | |
| Present | 15 | 8 (15.4) | 7 (13.5) | 61.5 (31.6–86.1) | 82.1 (66.5–92.5) | 0.41 | **0.001** | |
| Absent | 37 | 5 (9.6) | 32 (61.5) | 53.3 (26.6–79) | 86.5 (71.2–95.5) |  |  |  |
| Visual inattention/extinction defect # n=96 | | | | | | | | |
| Present | 19 | 14 (14.6) | 5 (5.2) | 78 (52.4–94) | 93.6 (85.7–98) | 0.70 | **<0.001** | |
| Absent | 77 | 4 (4.2) | 73 (76) | 74 (49–91) | 95 (87.2–98.6) |  |  |  |
| Visual inattention/extinction defect # Same day n=44 | | | | | | | |  |
| Present | 6 | 5 (11.4) | 1 (2.3) | 71.4 (29–96.3) | 97.3 (85.8–99.9) | 0.73 | **<0.001** | |
| Absent | 38 | 2 (4.5) | 36 (81.8) | 83.3 (36–99.6) | 95 (82.3–99.4) |  |  |  |
| Visual inattention/extinction defect # Different day n=52 | | | | | | | |  |
| Present | 13 | 9 (17.3) | 4 (7.7) | 82 (48.2–97.7) | 90.2 (77–97.3) | 0.67 | **<0.001** | |
| Absent | 39 | 2 (3.8) | 37 (71.2) | 69.2 (38.6–91) | 95 (83–99.4) |  |  |  |
| Reading defect ** n=90 | | | | | | | | |
| Present | 14 | 10 (11.1) | 4 (4.4) | 59 (33–82) | 94.5 (86.6–98.5) | 0.57 | **<0.001** | |
| Absent | 76 | 7 (7.8) | 69 (76.7) | 71.4 (42–91.6) | 91 (82–96.2) |  |  |  |
| Reading defect ** Same day n=41 | | | | | | | | |
| Present | 6 | 4 (9.8) | 2 (4.9) | 66.7 (22.3–95.7) | 94.3 (80.8–99.3) | 0.61 | **<0.001** | |
| Absent | 35 | 2 (4.9) | 33 (80.5) | 66.7 (22.3–95.7) | 94.3 (80.8–99.3) |  |  |  |
| Reading defect ** Different day n=49 | | | | | | | | |
| Present | 8 | 6 (12.2) | 2 (4.1) | 54.5 (23.4–83.3) | 95 (82.3–99.4) | 0.54 | **<0.001** | |
| Absent | 41 | 5 (10.2) | 36 (73.5) | 75 (35–97) | 88 (74–96) |  |  |  |

* V-FAST against orthoptic assessment; orthoptic assessment is the reference standard. † Defined as any visual defect in any section of orthoptic assessment and any visual defect in any section of the V-FAST, ‡ 2 missing, § Defined as any visual defect in eye movement section of orthoptic assessment and any visual defect in eye movement section of the V-FAST, ‖ Defined as any visual defect in visual fields section of orthoptic assessment and any visual defect in visual fields section of the V-FAST, # Defined as any visual defect in visual inattention/extinction section of orthoptic assessment and any visual defect in visual inattention/extinction section of the V-FAST, ** Defined as any visual defect in reading section of orthoptic assessment and any visual defect in reading section of the V-FAST. Bold fonts indicate statistical significance.

**Supplementary Table 8.** Subgroup analysis of performance and agreement on visual impairment detection of V-FAST versus orthoptic assessment in **stroke** population stratified by same or different dates.

| V-FAST  Orthoptic | Visual impairment | | | Diagnostic measure * | | | (V-FAST vs orthoptic) | | |
| --- | --- | --- | --- | --- | --- | --- | --- | --- | --- |
|  | **Total** | **Present**  **n (%)** | **Absent**  **n (%)** | **Sensitivity (95% CI)** | | **Specificity (95% CI)** | ***Kappa coefficient*** | ***P* value** | |
|  |  |  |  | **Positive predictive value (95% CI)** | **Negative predictive value (95% CI)** | |  |  |  |
| Overall † n=83 | | | | | | | | | |
| Any defect present | 53 | 25 (30.2) | 28 (33.7) | 92.6 (75.7–99.1) | | 50 (36.3–63.7) | 0.34 | **0.0001** | |
| No defect on orthoptic | 30 | 2 (2.4) | 28 (33.7) | 47.2 (33.3–61.4) | | 93.3 (78–99.2) |  |  |  |
| Overall † Same day n=36 | | | | | | | | | |
| Any defect present | 21 | 9 (25) | 12 (33.3) | 90 (55.5–99.7) | | 53.8 (33.4–73.4) | 0.33 | **0.008** | |
| No defect on orthoptic | 15 | 1 (2.8) | 14 (38.9) | 43 (22–66) | | 93.3 (68.1–99.8) |  |  |  |
| Overall † Different day n= 47 | | | | | | | | | |
| Any defect present | 32 | 16 (34) | 16 (34) | 94.1 (71.3–99.9) | | 46.7 (28.3–65.7) | 0.34 | **0.002** | |
| No defect on orthoptic | 15 | 1 (2.2) | 14 (29.8) | 50 (32–68.1) | | 93.3 (68.1–99.8) |  |  |  |
| Eye movements defect § n= 81 | | | | | | | | | |
| Present | 34 | 8 (9.9) | 26 (32.1) | 61.5 (31.6–86.1) | | 61.8 (49.2–73.3) | 0.14 | 0.06 | |
| Absent | 47 | 5 (6.2) | 42 (51.8) | 23.5 (10.7–41.2) | | 89.4 (77–96.5) |  |  |  |
| Eye movements defect § Same day n= 36 | | | | | | | | | |
| Present | 14 | 0 | 14 (38.9) | 0 (0–97.5) | | 60 (42.1–76.1) | -0.05 | 0.79 | |
| Absent | 22 | 1 (2.8) | 21 (58.3) | 0 (0–23.2) | | 95.5 (77.2–100) |  |  |  |
| Eye movements defect § Different day n= 45 | | | | | | | | | |
| Present | 20 | 8 (17.8) | 12 (26.6) | 66.7 (35–90.1) | | 63.6 (45.1–79.6) | 0.25 | **0.03** | |
| Absent | 25 | 4 (8.9) | 21 (46.7) | 40 (19.1–64) | | 84 (64–95.5) |  |  |  |
| Visual fields defect ‖ n=78 | | | | | | | | | |
| Present | 23 | 13 (16.7) | 10 (12.8) | 72.2 (46.5–90.3) | | 83.3 (71.5–91.7) | 0.50 | **<0.001** | |
| Absent | 50 | 5 (6.4) | 50 (64.1) | 56.5 (34.5–76.8) | | 91 (80–97) |  |  |  |
| Visual fields defect ‖ Same day n=35 | | | | | | | | | |
| Present | 10 | 6 (17.1) | 4 (11.4) | 85.7 (42.1–99.6) | | 85.7 (67.3–96) | 0.61 | **0.001** | |
| Absent | 25 | 1 (2.8) | 24 (68.7) | 60 (26.2–88) | | 96 (79.6–100) |  |  |  |
| Visual fields defect ‖ Different day n=43 | | | | | | | | | |
| Present | 13 | 7 (16.3) | 6 (13.9) | 63.6 (30.8–89.1) | | 81.3 (63.6–92.8) | 0.42 | **0.002** | |
| Absent | 30 | 4 (9.3) | 26 (60.5) | 53.8 (25.1–81) | | 86.7 (69.3–96.2) |  |  |  |
| Visual inattention/extinction defect # n=79 | | | | | | | | | |
| Present | 18 | 13 (16.5) | 5 (6.3) | 76.5 (50.1–93.2) | | 92 (82.2–97.3) | 0.67 | **<0.001** | |
| Absent | 61 | 4 (5.1) | 57 (72.1) | 72.2 (46.5–90.3) | | 93.4 (84.1–98.2) |  |  |  |
| Visual inattention/extinction defect # Same day n=35 | | | | | | | | |  |
| Present | 6 | 5 (14.3) | 1 (2.9) | 71.4 (29–96.3) | | 96.4 (81.7–99.9) | 0.71 | **<0.001** | |
| Absent | 29 | 2 (5.7) | 27 (77.1) | 83.3 (36–99.6) | | 93.1 (77.2–99.2) |  |  |  |
| Visual inattention/extinction defect # Different day n=44 | | | | | | | | |  |
| Present | 12 | 8 (18.2) | 4 (9.1) | 80 (44.4–97.5) | | 88.2 (72.5–96.7) | 0.64 | **<0.001** | |
| Absent | 32 | 2 (4.5) | 30 (68.2) | 66.7 (35–90.1) | | 94 (79.2–99.2) |  |  |  |
| Reading defect ** n=72 | | | | | | | | | |
| Present | 14 | 10 (13.8) | 4 (5.6) | 71.4 (42–91.6) | | 93.1 (83.3–98.1) | 0.64 | **<0.001** | |
| Absent | 58 | 4 (5.6) | 54 (75) | 71.4 (42–91.6) | | 93.1 (83.3–98.1) |  |  |  |
| Reading defect ** Same day n=32 | | | | | | | | | |
| Present | 6 | 4 (12.5) | 2 (6.2) | 80 (28.4–99.5) | | 92.6 (75.7–99.1) | 0.67 | **0.0001** | |
| Absent | 26 | 1 (3.1) | 25 (78.2) | 66.7 (22.3–95.7) | | 96.2 (80.4–100) |  |  |  |
| Reading defect ** Different day n=40 | | | | | | | | | |
| Present | 8 | 6 (15) | 2 (5) | 66.7 (30–92.5) | | 93.5 (78.6–99.2) | 0.63 | **<0.001** | |
| Absent | 32 | 3 (7.5) | 29 (72.5) | 75 (35–97) | | 90.6 (75–98) |  |  |  |

* V-FAST against orthoptic assessment; orthoptic assessment is the reference standard. † Defined as any visual defect in any section of orthoptic assessment and any visual defect in any section of the V-FAST, § Defined as any visual defect in eye movement section of orthoptic assessment and any visual defect in eye movement section of the V-FAST, ‖ Defined as any visual defect in visual fields section of orthoptic assessment and any visual defect in visual fields section of the V-FAST, # Defined as any visual defect in visual inattention/extinction section of orthoptic assessment and any visual defect in visual inattention/extinction section of the V-FAST, ** Defined as any visual defect in reading section of orthoptic assessment and any visual defect in reading section of the V-FAST. Bold fonts indicate statistical significance.

**Supplementary Table 9.** Subgroup analysis of performance and agreement on visual impairment detection of V-FAST versus orthoptic assessment in **non-stroke** population stratified by assessments undertaken on the same or different dates.

| V-FAST  Orthoptic | Visual impairment | | | Diagnostic measure * | | (V-FAST vs orthoptic) | | |
| --- | --- | --- | --- | --- | --- | --- | --- | --- |
|  | **Total** | **Present**  **n (%)** | **Absent**  **n (%)** | **Sensitivity (95% CI)** | **Specificity (95% CI)** | ***Kappa coefficient*** | ***P* value** | |
|  |  |  |  | **Positive predictive value (95% CI)** | **Negative predictive value (95% CI)** |  |  |  |
| Overall † n= 20 | | | | | | | | |
| Any defect present | 12 | 8 (40) | 4 (20) | 89 (51.8–99.7) | 63.6 (30.8–89.1) | 0.51 | **0.0085** | |
| No defect on orthoptic | 8 | 1 (5) | 7 (35) | 66.7 (35–90.1) | 87.5 (47.3–99.7) |  |  |  |
| Overall † Same day n= 9 | | | | | | | | |
| Any defect present | 4 | 2 (22.2) | 2 (22.2) | 66.7 (9.4–99.2) | 66.7 (22.3–95.7) | 0.31 | 0.17 | |
| No defect on orthoptic | 5 | 1 (11.2) | 4 (44.4) | 50 (6.8–93.2) | 80 (28.4–99.5) |  |  |  |
| Overall † Different day n= 11 | | | | | | | | |
| Any defect present | 8 | 6 (54.5) | 2 (18.2) | 100 (54.1–100) | 60 (14.7–94.7) | 0.62 | **0.01** | |
| No defect on orthoptic | 3 | 0 | 3 (27.3) | 75 (35–96.8) | 100 (29.2–100) |  |  |  |
| Eye movements defect § n= 19 | | | | | | | | |
| Present | 9 | 3 (15.8) | 6 (31.6) | 60 (14.7–94.7) | 57.1 (29–82.3) | 0.14 | 0.25 | |
| Absent | 10 | 2 (10.5) | 8 (42.1) | 33.3 (7.5–70.1) | 80 (44.4–97.5) |  |  |  |
| Eye movements defect § Same day n= 9 | | | | | | | | |
| Present | 3 | 2 (22.2) | 1 (11.1) | 66.7 (9.4–99.2) | 83.3 (35.9–99.6) | 0.50 | 0.07 | |
| Absent | 6 | 1 (11.1) | 5 (55.6) | 66.7 (9.4–99.2) | 83.3 (36–99.6) |  |  |  |
| Eye movements defect § Different day n= 10 | | | | | | | | |
| Present | 6 | 1 (10) | 5 (50) | 50 (1.3–99) | 37.5 (8.5–75.5) | -0.07 | 0.62 | |
| Absent | 4 | 1 (10 | 3 (30) | 16.7 (0.4–64.1) | 75 (19.4–99.4) |  |  |  |
| Visual fields defect ‖ n=18 | | | | | | | | |
| Present | 3 | 1 (5.6) | 2 (11.1) | 33.3 (0.8–90.6) | 86.7 (59.5–98.3) | 0.20 | 0.20 | |
| Absent | 15 | 2 (11.1) | 13 (72.2) | 33.3 (0.8–90.6) | 86.7 (59.5–98.3) |  |  |  |
| Visual fields defect ‖ Same day n= 9 | | | | | | | | |
| Present | 1 | 0 | 1 (11.1) | 0 (0–97.5) | 87.5 (47.3–99.7) | - 0.12 | 0.64 | |
| Absent | 8 | 1 (11.1) | 7 (77.8) | 0 (0–97.5) | 87.5 (47.3–99.7) |  |  |  |
| Visual fields defect ‖ Different day n= 9 | | | | | | | | |
| Present | 2 | 1 (11.1) | 1 (11.1) | 50 (1.3–98.7) | 85.7 (42.1–99.6) | 0.35 | 0.14 | |
| Absent | 7 | 1 (11.1) | 6 (66.7) | 50 (1.3–98.7) | 85.7 (42.1–99.6) |  |  |  |
| Visual inattention/extinction defect # n= 17 | | | | | | | | |
| Present | 1 | 1 (5.9) | 0 | 100 (2.5–100) | 100 (79.4–100) | 1 | **<0.001** | |
| Absent | 16 | 0 | 16 (94.1) | 100 (2.5–100) | 100 (79.4–100) |  |  |  |
| Visual inattention/extinction defect # Same day n= 9 | | | | | | | |  |
| Present | 9 | 9 (100) | 0 | - | - | - | - | |
| Absent | 0 | 0 | 0 | - | - |  |  |  |
| Visual inattention/extinction defect # Different day n= 8 | | | | | | | |  |
| Present | 1 | 1 (12.5) | 0 | 100 (2.5–100) | 100 (59–100) | 1 | **0.002** | |
| Absent | 7 | 0 | 7 (87.5) | 100 (2.5–100) | 100 (59–100) |  |  |  |
| Reading defect ** n= 18 | | | | | | | | |
| Present | 0 | 0 | 0 | - | - | - | - | |
| Absent | 18 | 3 (16.7) | 15 (83.3) | - | - |  |  |  |
| Reading defect ** Same day n= 9 | | | | | | | | |
| Present | 9 | 1 (11.1) | 8 (88.9) | - | - | - | - | |
| Absent | 0 | 0 | 0 | - | - |  |  |  |
| Reading defect ** Different day n= 9 | | | | | | | | |
| Present | 0 | 0 | 0 | - | - | - | - | |
| Absent | 9 | 2 (22.2) | 7 (77.8) | - | - |  |  |  |

* V-FAST against orthoptic assessment; orthoptic assessment is the reference standard. † Defined as any visual defect in any section of orthoptic assessment and any visual defect in any section of the V-FAST, § Defined as any visual defect in eye movement section of orthoptic assessment and any visual defect in eye movement section of the V-FAST, ‖ Defined as any visual defect in visual fields section of orthoptic assessment and any visual defect in visual fields section of the V-FAST, # Defined as any visual defect in visual inattention/extinction section of orthoptic assessment and any visual defect in visual inattention/extinction section of the V-FAST, ** Defined as any visual defect in reading section of orthoptic assessment and any visual defect in reading section of the V-FAST. Bold fonts indicate statistical significance.

**Supplementary Table10.** Patient-specific discordant results of V-FAST abnormal results versus normal orthoptic assessment (n=21).

| **Reason for discrepancy** | **Category as per V-FAST** | **Difference in time assessments performed** |
| --- | --- | --- |
| **V-FAST:** All the right side | VF | 4 days later  (Possible full recovery) |
| **V-FAST:** Right | VF | 3 days later  (Possible full recovery) |
| **V-FAST:** Abnormal | VF | 4 days later  (Possible full recovery) |
| **V-FAST:** Visual field (upper right)  **Orth:** Pupils defect and low vision assessment | VF | 5 hours later  (Partial recovery) |
| **V-FAST:** Right  **Orth:** Defect appears symmetrical not attending right | VF | 2 days later  (Possible partial recovery) |
| **V-FAST:** Abnormal | VF | 2 days later  (Possible full recovery) |
| **V-FAST:** Abnormal (right upper and lower) and intention to the right side | VF  VN | 20 hours later  (Possible full recovery) |
| **V-FAST:** Abnormal | VF  Reading | 15 hours later  (Possible full recovery) |
| **V-FAST:** Abnormal | Reading | 18 hours later  (Possible full recovery) |
| **V-FAST:** Abnormal | Reading | 6 days later  (Possible full recovery) |
| **V-FAST:** Abnormal | Reading | 5 days later  (Possible full recovery) |
| **V-FAST:** Nystagmus on eye movement upwards. | OM | 3 days later  (Possible full recovery) |
| **V-FAST:** Global nystagmus, eye movement (nystagmus)  **Orth:** Convergence not performed; but rest of eye movement elements are normal. | OM | 6 hours later  (Possible full recovery) |
| **V-FAST:** Not smooth movements to right, and nystagmus. | OM | 2 days later  (Possible full recovery) |
| **V-FAST:** Nystagmus | OM | 2 days later  (Possible full recovery) |
| **V-FAST:** Not smooth movements to left. | OM | 4 days later  (Possible full recovery) |
| **V-FAST:** Not smooth movements upwards, right, left and downwards | OM | 2 days later  (Possible full recovery) |
| **V-FAST:** Not smooth movements to left and downwards. Inattention to left side | OM  VN | 23 hours later  (Possible full recovery) |
| **V-FAST:** Eye alignment, not smooth movements to upwards, right, left and downwards. Eye movement, reading, visual fields (patients left), visual inattention | OM  VF  VN | 4 days later  (Possible full recovery) |
| **V-FAST:** Left visual inattention | VN | 12 hours later  (Possible full recovery) |
| **V-FAST:** No new visual or eye problem, previously right eye neglect.  **Orth:** Struggle to perform saccades testing, lower right quadrantanopia  << [Defect lower right quadrantanopia)].>> | VN | 17 hours later |

Orthoptic assessment is normal unless otherwise stated. Obs, Observations; OM, Ocular motility; VF, Visual fields; VN, Visual neglect.

**Supplementary Table 11.** Proportions of types of visual impairment in patient-specific discordant results of V-FAST abnormal results versus normal orthoptic assessment (n=21).

| **Discrepancy** | **N (%)**  **(n=21)** |
| --- | --- |
| **Any ocular motility with other abnormality** | **8 (38.1)** |
| *Ocular motility only* | 6 (28.7) |
| *Ocular motility & visual neglect* | 1 (4.7) |
| *Ocular motility, visual field & visual neglect* | 1 (4.7) |
| **Visual field** | **8 (38.1)** |
| *Visual field only* | 6 (28.7) |
| *Visual field & visual neglect* | 1 (4.7) |
| *Visual field & reading* | 1 (4.7) |
| **Reading** | **3 (14.3)** |
| **Visual neglect** | **2 (9.5)** |

**Supplementary Table 12.** Patient-specific discordant results of normal V-FAST results versus orthoptic assessment (n=44).

| **Reason for discrepancy** | **Category as per orthoptist assessment** | **Difference in time assessments performed** |
| --- | --- | --- |
| **Orth:** Saccades (horizontal & vertical) and pupils defect | Obs  OM | 27 days later |
| **Orth:** Smooth pursuits (horizontal & vertical; not following) and pupils defect | Obs  OM | 18 hours later |
| **Orth:** Eye movement, pupils defect, limited examination due to confusion | Obs  OM | 6 days later |
| **Orth:** Reduced convergence, pupils defect, lid defect and “Nil stroke related visual concerns” | Obs  OM | 3 days later |
| **Orth:** Eye movement, smooth pursuits (horizontal & vertical), saccades (horizontal & vertical), reduced convergence and pupils defect. | Obs  OM | 3 days later |
| **Orth:** Eye movement, smooth pursuits (horizontal & vertical) and lids defect | Obs  OM | 15 hours later |
| **Orth:** Eye movement, bilateral lid retraction (left > right) and lid tremor. | Obs  OM | 13 hours later |
| **Orth:** Eye movement, smooth pursuits (horizontal & vertical) and pupils defect. | Obs  OM | 1 day later |
| **Orth:** Eye movement, smooth pursuits (horizontal & vertical), saccades (horizontal & vertical), reduced convergence and pupils defect. (Cataract progression, no hemianopia on discharge) | Obs  OM | 4 days later |
| **Orth:** Eye movement, smooth pursuits (horizontal & vertical), saccades (horizontal & vertical), reduced convergence and pupils defect. | Obs  OM | 2 hours later |
| **Orth:** Eye movement, smooth pursuits (horizontal) and lids defect. | Obs  OM | 19 hours later |
| **Orth:** Eye movement, smooth pursuits (horizontal & vertical) and lids defect. | Obs  OM | 21 hours later |
| **Orth:** Eye movement and lids defect | Obs  OM | 3 hours later |
| **Orth:** Reduced convergence and right squint | OM | 16 hours later |
| **Orth:** Eye movement, smooth pursuits (horizontal & vertical) | OM | 3 days later |
| **Orth:** Eye movement, smooth pursuits (horizontal & vertical) | OM | 1 day later |
| **Orth:** Eye movement, saccades (horizontal & vertical) and reduced convergence | OM | 1 day later |
| **Orth:** Eye movement and smooth pursuits (horizontal + vertical). | OM | 5 hours later |
| **Orth:** Eye movement and absent convergence. | OM | 2 days later |
| **Orth:** Eye movement and smooth pursuits (horizontal & vertical). | OM | 2 days later |
| **Orth:** Eye movement and smooth pursuits (horizontal & vertical). | OM | 5 days later |
| **Orth:** Eye movement, smooth pursuits (horizontal & vertical), saccades (horizontal & vertical), pendular nystagmus (left > right)  << [pendular nystagmus (left > right) (Oscillopsia)]. >> | OM | 14 hours later |
| **Orth:** Absent convergence. | OM | 8 hours later |
| **Orth:** Eye movement, smooth pursuits (horizontal & vertical), saccades (horizontal & vertical) and absent convergence. | OM | 4 days later |
| **Orth:** Eye movement, reduced vision (right > left) | OM  VA | 1 day later |
| **Orth:** Eye movement, smooth pursuits (horizontal & vertical), Amblyopic right eye. No other issue. | OM  VA | 17 hours later |
| **Orth:** Eye movement, right inattention, abnormal alignment | OM  VN | 15 hours later |
| **Orth:** Absent convergence and right inattention | OM  VN | 13 hours later |
| **Orth:** Reduced convergence, right visual Inattention and visual fields | OM  VN  VF | 3 days later |
| **Orth:** Eye movement, smooth pursuits (horizontal & vertical), and visual fields (pre-existing eye history – glaucoma) | OM  VF | 3 days later |
| **Orth:** (Patient agitated and confused) Eye movement, smooth pursuits (horizontal & vertical), saccades (horizontal & vertical), reduced convergence and right hemianopia.  << [Right homonymous hemianopia].>> | OM  VF | 9 days later |
| **Orth:** Reduced convergence, right homonymous hemianopia and Alexia | OM  VF  Reading | 13 hours later |
| **Orth:** Reading, eye movement, smooth pursuits (horizontal), reduced convergence, visual fields Right (left upper and lower) Left (left upper and lower), complete left hemianopia,  << [Left hemianopia (complete)].>> | OM  VF  Reading | 1 day later |
| **Orth:** Eye movement, smooth pursuits (horizontal & vertical), saccades (horizontal & vertical), and reduced convergence. 0.3 Radner | OM  Reading | 2 days later |
| **Orth:** Partial Left hemianopia | VF | 2 days later |
| **Orth:** Left homonymous hemianopia | VF | 7 days later |
| **Orth:** Partial left hemianopia | VF | 10 days later |
| **Orth:** Lower left quadrants defect | VF | 2 days later |
| **Orth:** Visual fields and glaucomatous | VF | 17 hours later |
| **Orth:** Lower right quadrantanopia | VF | 17 hours later |
| **Orth:** Very mild left neglect | VN | 2 days later |
| **Orth:** Reading not possible – dysarthria and possible mild right neglect | Reading  VN | 1 day later |
| **Orth:** Reading, partial left hemianopia, left inattention  << [Mild left neglect. recovered partially left hemianopia quadrantanopia defect)].>> | Reading  VF  VN | 2 days later |
| **Orth:** Right lazy eye and left macular issue (longstanding pre-existing); pre-existing eye issue both eyes | VA | 14 hours later |

All V-FAST is normal. Obs, Observations; Orth, orthoptist; OM, Ocular motility; VF, Visual fields; VN, Visual neglect

**Supplementary Table 13.** Proportions of types of visual impairment in patient-specific discordant results of normal V-FAST results versus abnormal orthoptic assessment (n=44).

| **Discrepancy** | **N (%)**  **(n= 44)** |
| --- | --- |
| **Any ocular motility with other abnormality** | **34 (77)** |
| *Ocular motility only* | 11 (25) |
| *Observations & Ocular motility* | 13 (29.5) |
| *Ocular motility & visual acuity* | 2 (4.5) |
| *Ocular motility & visual neglect* | 2 (4.5) |
| *Ocular motility & visual field* | 2 (4.5) |
| *Ocular motility & reading* | 1 (2.2) |
| *Ocular motility, visual field & reading* | 2 (4.5) |
| **Visual field** | 6 (13.6) |
| **Visual neglect** | **3 (6.8)** |
| *Visual neglect only* | 1 (2.2) |
| *Visual neglect & reading* | 1 (2.2) |
| *Visual neglect, reading & visual neglect* | 1 (2.2) |
| **Visual acuity** | **1 (2.2)** |
